# A Case Illustrating the Efficacy of Dr. Ayre’s Treatment of Cholera

**Published:** 1849-11

**Authors:** 


*A Case illustrating the efficacy of Dr Ayre's treatment of Cholera.* By W. N. Spong, Esq., Surgeon, Feversham, Kent.

The Lancet has contained several valuable papers upon cholera, by Dr. Ayre. It appears to me that while the profession is casting about for new methods of treatment, we are already in possession of a most successful one, giving as high a per centage of recovery as the desperate nature of the disease can be reasonably expected to admit. The above-named gentleman recommends small doses of calomel and opium every few minutes, so long as the stage of collapse continues; and his extraordinary success is confirmed by competent eye-witnesses. The following case was sufficiently formidable to fairly test its powers, and to those who are yet undetermined as to the treatment of this fearful malady, I can confidently recommend it, and will boldly affirm, that when tried at the bar of experience, it will not be found wanting.

Edward C——, aged thirty-seven, a whitesmith, spare habit of boby, for years past has enjoyed good health; accustomed to drink freely of porter. Has been employed of late at Sittingbourne, a few miles from this town, where several cases of cholera have occurred.

July 20th.—Had some pain about the umbilicus early this morning, and felt generally indisposed, but was able to work until noon. Three or four hours after this he failed rapidly, and was forthwith brought home. At seven P. M. I first saw him. Present state: the whole person of a livid blue color, cold and shrunken; severe cramps of all the extremities, of the legs especially; pulse small and quivering, constant vomiting, urinary and biliary secretions suppressed; has had above thirty rice-water evacuations within the last few hours; sense of constriction across the thorax; intellect clear; vox cholerica; collapse. *Treatment:* To take two grains of calomel and two drops of tincture of opium every ten minutes. This is Dr. Ayre's plan of treatment. In addition, to have a hot-air bath, and take ten drops of chloroform with every alternative dose of the medicine; also, a drachm of strong mercurial ointment to be placed in each axilla; this was rubbed in by the patient himself in consequence of his frequent spasmodic movements. Twenty-seven drops of chloroform are equal to five minims.

21st.—One A. M.: After six hours' treatment there is decided amendment; the attacks of cramp and vomiting are less severe, and the intervals prolonged. Extreme thirst, but if more than half an ounce of fluid is taken at one time, it is instantly rejected, commencing reaction. Omit the opiate and chloroform. One P. M.: Improving; reaction fully established; the purging has ceased; cramps and vomiting only occasionally recur. To take the calomel every half hour.

22d.—At six this morning a well-marked crisis took place. The urinary secretion was restored after thirty-eight hours' suspension, the liver performed its function, and a black-colored motion was passed, followed by several characteristic chopped spinach stools. From this hour he gradually progressed. To take small doses of solution of acetate of ammonia with the alkali slightly in excess, and no other medicine.

27th.—Convalescent.

*Remarks.*—If you desire a particular remedy to be given, tell the nurse to give it; but if you want it to be given properly, give it yourself. The latter part of this precept was acted upon for the first eight hours, and the medicine punctually given. The readiest plan is, to throw a couple of drachms of calomel on a sheet of paper, and divide and sub-divide into sixty parts; one of those for a dose is best taken from the point of a table knife. Again, with regard to the opiate, bale out any number of spoonfuls of water, add twice the number of drops of laudanum, using this spoon to administer the same; thus accurate quantities are known to be given. A hot-air bath is easily and efficiently given as follows:—Fasten a blanket loosely around the body with large pins, to prevent the patient kicking it off during the attacks of cramp; place two chairs upon the bed, so that the backs form an arch over the person, tie them side by side, and over these throw several other blankets. A spirit-lamp can now be safely held beneath at the foot for a few minutes, which will suffice to raise the temperature to one hundred and thirty degrees or more. In this disease minutes are as valuable as hours; if a spirit-lamp is not at hand, an excellent one can be made out of a tea-pot; pour in four or five ounces of spirits of wine, and made a wick by unravelling a piece of calico, collect the threads into a twist form, pass these down the spout, and the lamp is perfect. The hot-air bath appeared to relieve the internal congestion, and the chloroform was given with the intention of mitigating the agonizing cramps. But supposing the question to be fairly put, "What was *the* remedy in this case?"—my answer would be, most decidedly, the calomel, given in this particular manner. The patient took two drachms and a half of this, about one drachm of chloroform, and only forty minims of tincture of opium; the gums were but slightly touched. No brandy was at any time given, and to this is in great part attributed the entire absence of consecutive fever. During the collapse a circumstance occurred that deeply affected this man; but I observed, that although he cried, or, more strictly speaking, made a noise, yet he could not shed tears; this secretion, in common with all others, wanting its healthy stimulus. At the present moment it behooves every practitioner to be fully prepared in his own mind as to the method of treatment he intends to pursue, when called upon to combat this terrific enemy. If the constitution is not utterly depraved, Dr. Ayre has ably shown that calomel may be depended upon, and with this for a sheet anchor, the frail vessel will often outride the storm.—*London Lancet.*

